# Intra-arterial Infusion of Autologous Bone Marrow Mononuclear Stem Cells in Subacute Ischemic Stroke Patients

**DOI:** 10.3389/fneur.2016.00228

**Published:** 2016-12-16

**Authors:** Azza Abass Ghali, Mohamed Khalil Yousef, Osama AbdAllah Ragab, Enas Arafa ElZamarany

**Affiliations:** ^1^Neuropsychiatry, Tanta University, Tanta, Egypt; ^2^Clinical Pathology, Tanta University, Tanta, Egypt

**Keywords:** stem cell, ischemic stroke, intra-arterial, BM-MNC, safety

## Abstract

**Introduction:**

Based on many preclinical and small clinical trials, stem cells can help stroke patient with the possibility of replacing the cells and supporting the remaining cells. The aim of this study was to evaluate the safety and feasibility of bone marrow mononuclear (BMMN) stem cell transplantation in subacute ischemic stroke patients.

**Materials and methods:**

Thirty-nine (*n* = 39) patients with subacute ischemic cerebral infarct due to large artery occlusion in the middle cerebral artery (MCA) territory were recruited. They were distributed into two groups: first group (*n* = 21) served as an experimental group, which received intra-arterial (IA) mononuclear stem cells (bone marrow-derived mononuclear cell), while the other group (*n* = 18) served as a control group. All the patients were evaluated clinically by National Institutes of Health Stroke Scale, modified Rankin Scale, Barthel Index, modified and standardized Arabic version of the Comprehensive Aphasia Test, and radiological for 12 months.

**Results:**

The stem cell-treated group showed better improvement, but it was not significant when compared with the non-treated group. The volume of infarction changes at the end of the study was non-significant between both the groups. There was no, or minimal, adverse reactions in stem cell-treated group.

**Conclusion:**

The study results suggest that autologous BMMN stem cell IA transplantation in subacute MCA ischemic stroke patients is safe with very minimal hazards, but no significant improvement of motor, language disturbance, or infarction volume was detected in stem cell-treated group compared with the non-treated group.

## Introduction

Stroke represents the third cause of death, followed by cancer and myocardial infarction. Its morbidity and mortality keep increasing during the past few years, especially in developing countries (1).

The prevalence of non-fatal stroke in Egypt is 5.6 per 1,000 populations (2), and the disability-adjusted life years (the sum of life years lost as a result of premature death and years lived with disability adjusted for severity lost per 1,000 population) was 8 in 2003 (3).

Despite the availability of active therapies as thrombolytic therapy and percutaneous intravascular interventions, many patients suffering from stroke often remain disabled (4). These post-stroke disabilities have attracted the attention of researchers to explore more effective and safer treatments. Stem cells offer the promise of a novel neurorestorative strategy for acute brain injury. Recent preclinical studies in rodent stroke model using stem cell therapy have demonstrated significant behavioral recovery and, in some cases, reductions in lesion volume (5).

Although there are many types of stem cells, bone marrow-derived mononuclear cells (BM-MNCs) represent one of the most convenient types for clinical use, as they can be isolated rapidly from autologous bone marrow without culture (6).

There are many potential mechanisms of cellular therapy by which transplanted stem cells are able to locate to the infarcted brain area and differentiate into neuronal and glial phenotypes; there is evidence that cell replacement could contribute to the reestablishment of neuronal circuits by increasing the sprouting of nerve fibers (7).

In the experimental model of stroke, intravenous, intra-striatal, and intra-arterial (IA) infusion of mononuclear stem cells have improved neurological outcome through reduced apoptosis and decreased peri-infarct inflammation and angiogenesis (8).

The aim of this study is to assess the safety and feasibility of autologous bone marrow mononuclear (BMMN) stem cells in managing patients suffering from large artery ischemic stroke in the territories of middle cerebral artery (MCA) in the subacute stage. This study was approved by the ethical committee of Faculty of Medicine, Tanta University, Egypt.

## Patients and Methods

Thirty-nine patients with ischemic cerebral infarction due to large artery occlusion in the MCA territories in the subacute stage (1 week and up to 3 months) were enrolled in this study. None of them had the chance to receive thrombolytic therapy as an early treatment of ischemic stroke, and they were encoded by numbers to maintain their privacy.

Patients were distributed (according to their choice) into one of the two groups: the first group consisted of stem cell-treated patients (SCTPs), which included 21 patients who received autologous BMMN stem cell by IA (internal carotid) infusion ipsilateral to the infarction side at one session and continued on medical treatments according to the stroke guidelines of American Heart Association/American Stroke Association (9). The second group consisted of non-stem cell-treated patients (NSCTPs), which included 18 patients who served as control received medical treatments according to the stroke guidelines (9). An informed consent was obtained from all the participants or their first-degree relatives.

Patients enrolled in this study had ischemic stroke lasting for more than 1 week and less than 3 months; the neuro-images showed cerebral infarct in the MCA territory. Their National Institutes of Health Stroke Scale (NIHSS) between 4 and 20 and spontaneous re-canalization of the MCA were documented using transcranial Doppler.

Patients with cardio-embolic stroke, ASPECT score less than 5/10, severe carotid stenosis (>70%, by Doppler), primary hematological disease, neurodegenerative disorder, previous stroke with modified Rankin Scale (mRS) >2, auto-immune disorders, liver failure, chronic renal failure, or lacunar stroke were excluded from the study.

All the participants were evaluated by detailed medical history, physical examination, and neurological assessment that included the NIHSS, mRS, and Barthel index (BI). Patients with language disturbance were assessed by modified and standardized Arabic version of the Comprehensive Aphasia Test (10).

Neuro-imaging including brain CT or MRI to confirm ischemic stroke lesion site and excluding other intracranial pathology were obtained. Carotid system was assessed by linear array transducer of multi-frequency (3–12 MHz), real-time, sagittal, coronal, and axial views, while intracranial vessels were assessed by phased array transducer of multi-frequency (1–3 MHz), trans-axial, mesencephalic view through temporal window.

Laboratory tests including complete blood count and biochemical tests for urea, creatinine, electrolytes, fasting and postprandial blood sugar, coagulation profile, and serum cholesterol and triglyceride were performed.

Stem cell-treated patients were submitted to bone marrow aspiration, which is a standard practice at clinical pathology department in molecular diagnosis of hematological disease, Tanta University Hospital, which has been in use for many years. Three days before the procedure, patients received a daily SC injection of granulocyte colony stimulating factor (Pegfilgrastim) (Geneleukim^®^) (G-CSF at 300 μg/vials).

After aspiration of 50–100 ml of the BM, they were diluted at a ratio of 2:1 with clinical buffer; then, bone marrow filter was wetted with clinical buffer before use, and then, the diluted BM passed through it using sterile Pasteur tubes. Then, 35 ml of the diluted cell suspension was carefully layered over 15 ml of ficoll paque in a 50-ml conical tube and centrifuged at 2,000 rpm for 20 min at 20°C in a swinging out bucket rotor without brake. The interphase cells (mononuclear cells including SC and thrombocytes) were carefully transferred to a new 50-ml conical tube.

Cell pellet was washed by adding up to 40 ml of clinical buffer, mixed gently, and centrifuged at 1,200 rpm for 15 min at 20°C. Then, the supernatant was carefully and completely removed (this step was done twice) and finally cell pellet was suspended in an appropriate amount of clinical buffer.

The IA infusion was carried out a few minutes after separation of bone marrow SC using a Toshiba digital subtraction angiography (DSA). Patients were brought to the angiography suite and placed in the supine position, and an 18- or 20-gauge IV line is started. Ascending through the RT femoral artery to the aorta by diagnostic catheter and guiding hydrophilic J-shaped wire to reach the aortic arch, and from there, we select either right or left common carotid artery, then the internal carotid artery, and begin infusion of stem cells. After infusion of about 1 × 10^6^ cells suspended in 100-ml saline, the catheters and sheaths were removed, and hemostasis was achieved by either manual compression or with the use of a closure device.

All SCTPs were evaluated every hour for the first 24 h for vital signs, any neurological deterioration, and anaphylactic reaction, and then every day for the following week.

All patients in both groups were followed up monthly for neurological assessment using NIHSS, mRS, and BI, and patients with language disturbances were assessed by modified and standardized Arabic version of the Comprehensive Aphasia Test.

Neuro-imaging including brain CT to follow volume changes of infract area was carried out every 4 months for 1 year. For the assessment of volume of infarction, we used Toshiba Aquilion 16 CT Scanner^®^. The images were displayed and analyzed *via* DICOM 3.0 standards software. Using the manual tracing technique (this was carried out by trained radiologist, blind to cases), the perimeter of the area of abnormal low attenuation was traced on each CT slice showing the infarct, and the software automatically calculates the surface area being traced. The sum of all areas traced in all CT slices multiplied by the thickness of the slice is the infarction volume.

### Statistical Analysis

Statistical presentation and analysis of the present study was conducted, using the mean, SD, and chi-square test by SPSS V.16. In all of the statistical analysis, *p* < 0.05 was regarded as statistically significant.

## Results

Twenty-one patients were included in the SCTPs group (12 males and 9 females with their age ranged between 46 and 66 years). While NSCTPs consisted of 18 patients (10 males and 8 females with their age ranged between 48 and 65 years) who served as control and received regular treatments according to the stroke guidelines.

The earliest day of stem cells infusion was 12 days post-stroke onset, and the latest day was 32 days post-stroke onset with a mean of 22 days post-stroke onset.

**Figure 1:**
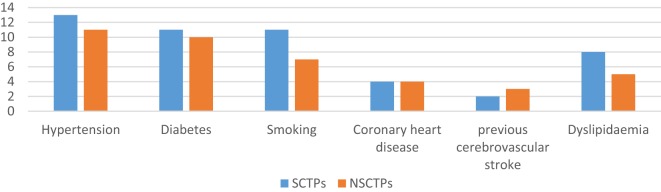
**Baseline medical condition, comorbidities, and risk factors of stroke between both groups**.

There were no significant differences between the groups with respect to comorbidities and risk factors of stroke (Figure 1). There were 24 patients with hypertension (61.9% of SCTP group and 61.1% of NSCTP group), 21 patients with diabetes mellitus (52.4% of SCTP group and 55.6% of NSCTP group), 18 patients were smokers (52.4% of SCTP group and 38.9% of NSCTP group), 8 patients had coronary artery disease (19% of SCTP group and 22.2% of NSCTP group), 5 patients had previous cerebrovascular strokes (9.5% of SCTP group and 18.7% of NSCTP group), and 13 patients had dyslipidemia (38.1% of SCTP group and 27.1% of NSCTP group).

### Clinical Outcomes

**Figure 2:**
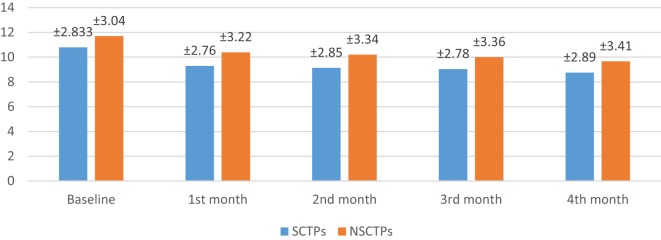
**Follow-up of both groups by National Institutes of Health Stroke Scale**.

**Figure 3:**
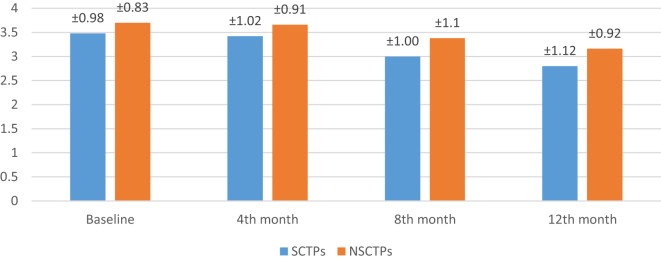
**Follow-up of both groups by modified Rankin Scale**.

At the beginning of this study, the NIHSS was not significant in both groups (*p* = 0.364). At the fourth month of the follow-up, we found significant improvement in NIHSS within each group, but this improvement was not statistically significant on comparison (*p* = 0.376; Figure 2). The initial mRS was not significant in both groups (*p* = 0.452), while at the end of the study, both groups showed significant improvement in mRS, without statistical significance between them (*p* = 0.290; Figure 3).

**Figure 4:**
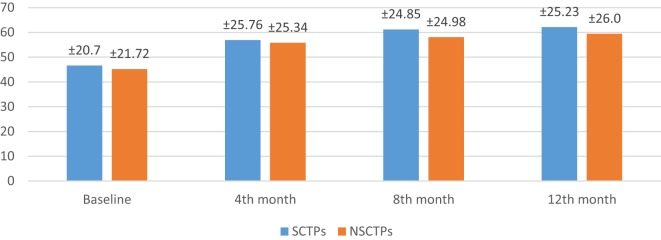
**Follow-up of both groups by Barthel Index**.

**Figure 5:**
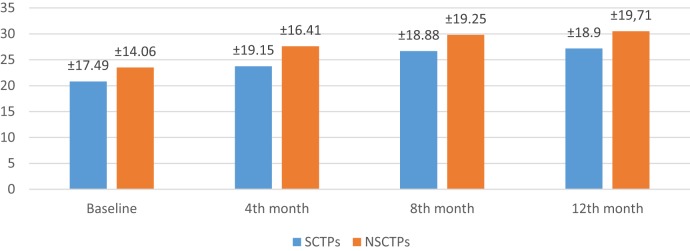
**Follow-up of both groups by modified and standardized Arabic version of the Comprehensive Aphasia Test**.

Also, the baseline BI was not different in both groups (*p* = 0.84), while at 12 months of follow-up, we found statistically significant improvement in BI within each group, but this improvement was not statistically significant on comparison (*p* = 0.745) (Figure 4). The language deficit was assessed by modified and standardized Arabic version of the Comprehensive Aphasia Test, which was not significant in both groups at the beginning of the study (*p* = 0.513). At the end of the study, we found significant improvement within each group, without significant difference between both groups (*p* = 0.691) (Figure 5).

### Radiological Outcomes

**Figure 6:**
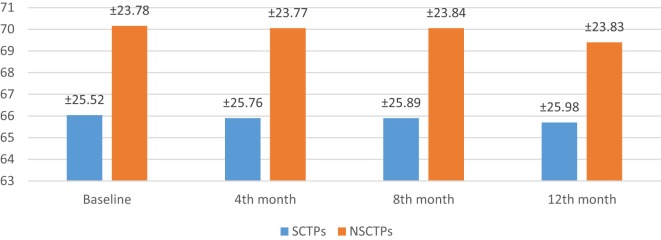
**The changes of infarction volume between both groups**.

At the beginning of this study, the difference of volume of infarction was not significant in both groups (*p* = 0.606). At the end of the study, the changes in the volume of infarction between both groups were minimal (*p* = 0.602); even within each group separately, there was no significant changes from baseline (Figure 6).

### Safety and Trial Conduct

In our study, two patients developed ecchymosis at the site of BM aspiration (9.5%) and they did not need any intervention. Mild pain was present in all patients after several hours of BM aspiration (100%), which was relieved totally with a single dose of NSAIDs.

One of our patients died 5 months post-procedure due to severe chest infection followed by respiratory failure and death. None of our patients developed either systemic or intracranial infection, embolic complication, seizure, or tumor formation during the study period. One patient in the SCTPs group developed mild renal impairment 3 months post-procedure, which improved on conservative treatment. One patient in NSCTPs group developed recurrent cerebrovascular stroke, and another patient developed mild renal impairment, which improved on conservative treatment.

## Discussion

A major advantage of cell therapy is that it will open the therapeutic time window of ischemic stroke such that a larger patient population will get benefit. This time window is 3 to 6h longer than that required for thrombolytic therapy (11). Preclinical studies demonstrated that the use of bone marrow stem cells had beneficial outcome in behavioral improvement in a rat stroke model. However, translating this early success into clinical applications was difficult due to both the limitation in source tissue and the ethical concerns (12–14).

When we started on designing this study, we put in our consideration that stroke is a heterogeneous disease that typically affects elderly people with significant comorbidities such as hypertension, atherosclerosis, cardiac disorders, and diabetes mellitus. In addition, female hormonal profile may play role as risk factors for stroke and affect response to treatment. So, to avoid all these variances affect our study results, we recruited 39 patients with ischemic cerebral infarct due to large artery occlusion in the MCA territories in subacute stage; none of them had the chance to receive thrombolytic therapy as early treatment of ischemic stroke, and this was intended to avoid good prognostic effect of tPA on clinical outcome.

In this study, only patients with partial MCA ischemic stroke were recruited and patients with ASPECT score less than 5 were excluded due to the expected bad prognosis, and theoretically, scaffolding and repeated doses of stem cell were required to cover this huge area of infarction.

Our results proofed safety of BMMN transplantation and agree with Prasad et al. (15) who found that all procedures were totally safe, as no serious adverse events were observed during their study. Also, Battistella et al. (16) assessed the safety and feasibility of IA transplantation of BMMN stem cells in six patients suffering from infarction in the MCA territories within 90 days of symptom onset; their results showed no stroke recurrence or cell-related adverse events during the procedure or during the follow-up period of 180 days.

The assessment of clinical outcome showed a functional improvement in both groups; however, the SCTPs showed better scores in NIHSS, mRS, and BI; this improvement was not statistically significant when compared to the NSCTPs. This finding was the same when we assessed language improvement.

The favorable outcomes were confirmed by the previous work of Prasad et al. (8), as they reported non-significant improvement in BI in their patient when compared to the control group. Also, Moniche et al. (17) reported non-significant changes of BI after IA BMMN stem cells infusion.

On the other hand, Bhasin et al. (18) reported statistically significant improvement in modified BI after 24 weeks between stem cell group and control group; however, they did not find significant changes in functional MR imaging in both groups. After we reviewed their patients and methods, we found that they infused both mononuclear and expanded mesenchymal stem cells intravenous in patients with ischemic and hemorrhagic stroke, which could explain the difference between our results and theirs.

Also, Suárez-Monteagudo et al. (19) reported a significant improvement in equilibrium, locomotion, and increased functional capacity in five patients who received intracerebral stem cell. Tthis conclusion could be explained by the small sample size, lack of control group, and recruiting patients with hemorrhagic stroke.

Lee et al. (20) reported improvement in mRS score after long-term follow-up (5 years) in patients who received IV mesenchymal stem cells when compared to the control group. When we revised their patients’ characteristic, we found that 12 patients received thrombolytic therapy during acute stroke, so we can claim that the improvement may be related to thrombolytic therapy and not related to stem cells effect.

Unfortunately, there were no clinical trials to assess language as an outcome of stem cell therapy in stroke patient. This is because most of the researchers were focusing on assessing safety as the primary outcome and motor deficit as the secondary outcome.

There is a case report by Bringas et al. (21), which reported a case of a 55-year-old Caucasian woman who received intracerebral autologous bone marrow stem cell 3 years after a subcortical stroke; she exhibited positive cognitive changes 6 months and 1 year after the surgery without rehabilitation. One year later, she showed improvement in mental flexibility, receptive language, phonological fluency, verbal memory, and auditory verbal memory.

Regarding the changes of infarction volume in both groups, our results showed no significant changes within each group even when comparing both groups. These results agreed with that of the previous work by Bhasin et al. (18); however, they reported that the stem cell group showed more functional improvement, but they did not find significant difference in functional brain MRI between experimental and control groups at 8 weeks and 6 months post-treatment. Also, Friedrich et al. (22) reported non-significant difference in infarct volumes or infarct densities between groups with and without clinical improvement.

Mendonça et al. (23) reported an improvement in the ischemic area assessed by MRI diffusion and in hypo-perfusion by SPECT 7 days post-mononuclear stem cell IA transplantation at 3 days post-stoke onset. But this was only a case report and the patient received cell therapy early on third day of admission, and this improvement in image may be a result of improvement, which was due to resolved brain edema and revascularization as stem cells need more time to differentiate into neuron or vascular endothelial cells.

In the present study, we carried out BMMN stem cell transplant in the early subacute stage. The procedure day after stroke onset is 22 ± 5.56. We selected this time window upon revision of preclinical trials (24, 25). Also, stroke patients are liable for deterioration in early days either by the effect of cerebral edema or hemorrhagic transformation. Finally, some patients with ischemic stroke have spontaneous recovery; so, we selected the subacute stage to be sure that the patients have stable deficit due to ischemic stroke.

On the contrary, in most of the preclinical studies, stem cells were given <24 h after ischemia (26). This is partly because of the opening of the blood–brain barrier after cerebral ischemia, which allows cells to enter the brain parenchyma. Also, the expression of various chemotactic signals peaks at this time point and guides the cells toward ischemic areas. However, while early cell transplantation may provide neuroprotection, the hostile environment endangers the long-term survival of transplanted cells. Transplantation at later time points may target the process of neurodegeneration and enhance the brain’s own repair mechanisms (27).

In this study, we selected the IA route as it involves endovascular infusion of stem cells directly in the artery perfusing the infarcted tissue. This route has the advantage of bypassing the peripheral filtering organs, thereby increasing the dose of cell delivered to the target tissue with uniform distribution.

One study comparing IV versus IA autologous BM-MNC infusion reported significant reduction in infarct volume, higher cell engraftment, and improved motor function in favor of the IA route. The authors attributed this “significant neuroprotective effect” in the IA group to the higher number of implanted cells in the brain during early reperfusion (28).

Finally, it has been hypothesized that infused stem cells were not only preferentially home in the infarcted brain but also secrete neurotrophic growth factors and act as “scaffolds” making the host environment appropriate for behavioral recovery and help endogenous neurogenesis after stroke. In addition to local effects on the damaged tissue, transplanted cells may help in recruiting progenitor cells from other tissues, as they could mobilize endogenous endothelial progenitors into circulation to enhance angiogenesis (29).

## Conclusion

Our results suggest that autologous BM-MN stem cell IA transplantation in subacute MCA ischemic stroke is safe with very minimal hazards, but no significant improvement of motor, language disturbance, or volume of infarction was detected in treated group when compared with the control group.

### Study Limitations

The limited sample size of patient and the absence of implanted cell tracking *in vivo* were the main limitations of this study.

### Recommendation

Large trials should be planned with stroke patients for getting appropriate decision about application of stem cells in clinical approach. Also, testing different types of stem cells, early injection of stem cells in the acute stage of stroke, and repeated booster doses of stem cell transplantation may be more beneficial than single injection. Finally, monitoring of survival, migration, and function of these cells by non-invasive imaging may reveal their fate in the host brain.

## Ethics Statement

Study approved by the Quality Assurance Unit, Research Ethics Committee, Faculty of Medicine Tanta University, approval number 593/05/11. The consent for participation in this study was signed by first degree relative in case of patients with language disturbance.

## Author Contributions

EE was responsible for stem cell separation and preparation. OR was responsible for recruiting patients’ clinical and radiological follow-up and shared intra-arterial stem cell infusion. MY was responsible for intra-arterial stem cell infusion. AG was responsible for recruiting patients’ clinical and radiological follow-up.

## Conflict of Interest Statement

The authors declare that the research was conducted in the absence of any commercial or financial relationships that could be construed as a potential conflict of interest.
